# Adaptive Disturbance Suppression Method for Servo Systems Based on State Equalizer

**DOI:** 10.3390/s24134418

**Published:** 2024-07-08

**Authors:** Jinzhao Li, Yonggang Li, Xiantao Li, Dapeng Mao, Bao Zhang

**Affiliations:** 1 University of Chinese Academy of Sciences, No. 19, Yuquan Rd., Beijing 100049, China; lijinzhao@ciomp.ac.cn; 2 Changchun Institute of Optics, Fine Mechanics and Physics, Chinese Academy of Sciences, Changchun 130033, China

**Keywords:** aviation optoelectronic stability platform, adaptive robust control, high frequency disturbance, state equalizer speed closed-loop

## Abstract

Disturbances in the aviation environment can compromise the stability of the aviation optoelectronic stabilization platform. Traditional methods, such as the proportional integral adaptive robust (PI + ARC) control algorithm, face a challenge: once high-frequency disturbances are introduced, their effectiveness is constrained by the control system’s bandwidth, preventing further stability enhancement. A state equalizer speed closed-loop control algorithm is proposed, which combines proportional integral adaptive robustness with state equalizer (PI + ARC + State equalizer) control algorithm. This new control structure can suppress high-frequency disturbances caused by mechanical resonance, improve the bandwidth of the control system, and further achieve fast convergence and stability of the PI + ARC algorithm. Experimental results indicate that, in comparison to the control algorithm of PI + ARC, the inclusion of a state equalizer speed closed-loop compensation in the model significantly increases the closed-loop bandwidth by 47.6%, significantly enhances the control system’s resistance to disturbances, and exhibits robustness in the face of variations in the model parameters and feedback sensors of the control object. In summary, integrating a state equalizer speed closed-loop with PI + ARC significantly enhances the suppression of high-frequency disturbances and the performance of control systems.

## 1. Introduction

**Figure 1:**
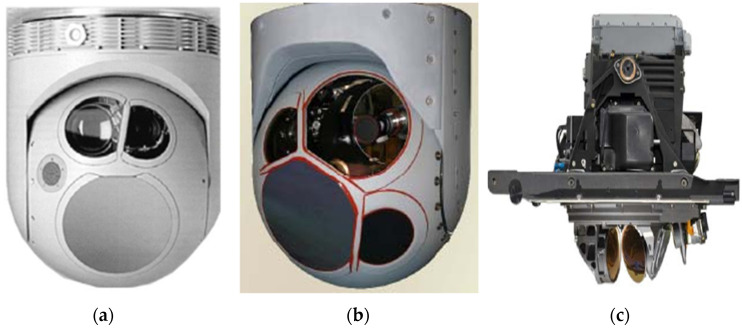
Several advanced aviation optoelectronic stabilization platforms. (**a**) AN/AAQ-30; (**b**) MX20; (**c**) EOTS.

As a crucial payload for unmanned aerial vehicles, the airborne optoelectronic stabilization platform serves as a core component for inertial navigation, guidance, and measurement. It is extensively employed in areas such as aviation reconnaissance, target positioning, strike calibration, battlefield damage assessment, and aerial surveying. Its primary function is to isolate carrier disturbances, ensuring a stable spatial orientation of the optical equipment’s line of sight and stable tracking of designated targets. Figure 1 shows several advanced aviation optoelectronic stabilization platforms.

**Figure 2:**
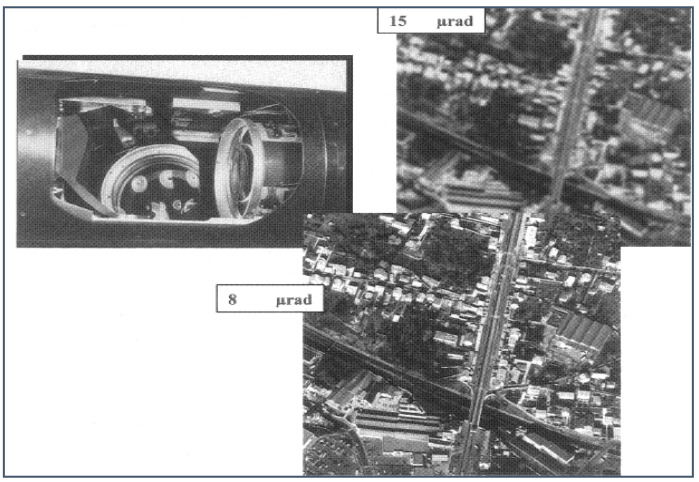
Images of the same target area with different visual axis stability accuracies.

As the detection range of the optical equipment mounted on the platform expands, the performance standards for airborne optoelectronic platforms are becoming increasingly stringent. The Formula (1) indicates that once the optical system is established, higher accuracy in line-of-sight stability leads to clearer imaging and more stable tracking. Consequently, enhancing the accuracy of line-of-sight stability is crucial for long-distance detection and reconnaissance using long focal lengths. Figure 2 presents a comparison of optical system imaging under varying line-of-sight stabilization accuracies achieved by the aviation optoelectronic stabilization platform integrated into the Global Hawk unmanned reconnaissance aircraft. As shown in the figure, the information images obtained from the 15 µrad and 8 µrad stable accuracy airborne optoelectronic platforms are shown. We can see that the information image clarity obtained by the 8 µrad stable accuracy airborne optoelectronic platform is significantly better than that obtained by the 15 µrad stable accuracy airborne optoelectronic platform.

(1) $$\sigma_{resolution\_ratio}=\sqrt{\sigma_{optical\_design}+\sigma_{\mathrm{detector}}+\sigma_{stabilization\_precision}+\sigma_{atmospheric\_transmissivity}+\cdots }$$

As is well known, under airborne conditions, due to the continuous changes in the aircraft’s flight attitude and interference from the external environment on the platform, geometric constraints and friction torque between the platform axis systems are directly coupled to the tracking equipment, resulting in a decrease in the stability accuracy of the sensor’s line of sight. In severe cases, it may even cause the tracking equipment to be unable to effectively track the target. It is evident that enhancing the system’s resistance to disturbances is a crucial aspect in improving the precision of the system’s line of sight stability. Consequently, the focus of current research is on methods to bolster the anti-interference and adaptive control capabilities of aviation optoelectronic stabilization platforms.

In the realm of interference suppression and adaptation, scholars and practitioners from various industrial sectors have conducted extensive research on relevant methods [1]. For instance, robust controllers can be designed to ensure good stability and control performance of the system in the presence of disturbances and model uncertainties. Common methods for disturbance suppression and adaptation include sliding mode control (SMC) [2,3,4], adaptive robust control (ARC) [5,6], H-infinity control [7,8], among others. The sliding mode control method is a robust nonlinear control strategy that ensures the system’s insensitivity and robustness to disturbances and uncertainties on the sliding surface [9]. In practical applications, the kinematic and dynamic models of robots are to some extent unknown. Ref. [10] studies an adaptive robust control method for fully constrained cable driven parallel robots, which does not require a predictive upper bound of system uncertainty and linear regression form, making it more flexible. Ref. [11] adopts a robust H-infinity output feedback controller to ensure the dynamic performance of the system under non holonomic constraints in the presence of model uncertainty and external disturbances. Ref. [12] uses sliding mode controllers and disturbance observers to control quadcopter drones with unmodeled disturbances. Ref. [13] establishes a hierarchical control strategy for suspension torque gyroscopes, which includes a state space based disturbance observer and an H-infinity robust controller. Researchers have successfully solved the control problem of active isolation systems under multiple constraint conditions through adaptive and robust design methods [14]. Adaptive robustness has also been used for model-based friction estimation and compensation, and to achieve precise position control of linear motors [15]. This type of adaptive robust control method can meet the required output error transient performance and final steady-state tracking accuracy even in the presence of both parameter uncertainty and uncertain nonlinearity. The adaptive robust control method has shown excellent performance in various fields such as high-precision position control and speed control [16,17]. However, the design objectives of these methods are focused on the convergence of errors between commands and responses, rather than purposeful disturbance compensation. Controller design must consider both the effects of interference and system performance. When the system encounters high-frequency disturbances due to mechanical resonance, the control effectiveness is limited.

In summary, high-frequency disturbances due to mechanical resonance significantly limit the bandwidth of servo control systems. This, in turn, impacts controller design, making it challenging to ensure satisfactory system stability. Consequently, numerous methods have been developed to suppress mechanical resonance.

In structural design, the system’s resonance frequency can be enhanced by adjusting the structural style to lower the load’s rotational inertia or by utilizing materials with greater rigidity. This increases the system’s bandwidth and enhances the controller’s response speed and interference suppression capabilities [18]. However, these methods not only raise design costs but also only marginally increase the mechanical resonance frequency without directly suppressing or resolving the issue. In the field of designing notch filters, to mitigate the phase lag associated with their use, scholars worldwide have conducted extensive research on their structures and control strategies. Ref. [19] proposes a design scheme for zero phase notch filter (ZPNF), which reduces the phase delay caused by the notch filter and improves the positioning speed of industrial robotic arms; Ref. [20] proposes a new type of ZPNF, which preprocesses and filters the position control signal. Experimental results demonstrate that the end effector jitter of the robotic arm is suppressed, and the tracking performance is enhanced; Ref. [21] proposes the zero phase error tracking controller (ZPETC) scheme based on ZPNF, which effectively integrates both to enhance the control performance of robotic arms. This approach was subsequently validated through simulation; Ref. [22] proposes a small phase error compensation strategy using a notch filter for the positioning and position tracking of robotic arms, which improves the tracking performance of the robotic arm. However, these designs primarily target the control of the position loop in servo systems, and the design of zero phase notch filters is complex. There is scant detailed research on reducing the control delay in speed loops caused by notch filters and enhancing the speed response beyond conventional second-order notch filters. In the realm of advanced control methodologies, Ref. [23] compares two adaptive control strategies: one employs a neuro-fuzzy sliding mode controller for direct control, while the other relies on a Kalman filter to measure system state variables for feedback control. However, the latter is only effective at resonant frequencies of approximately 10 Hz. The author highlights that both methods have their strengths and weaknesses, and the selection should be based on the specific circumstances. To suppress resonance while ensuring robust control, Ref. [24] employs an adaptive sliding neural fuzzy controller and a model reference-based adaptive control structure. In terms of applicability, this approach surpasses both PI-type neural fuzzy controllers and classic cascade PI control. Ref. [25] employs a genetic algorithm to tune the parameters of the PMSM speed controller PID. Kazuaki Itoh and colleagues designed a robust controller with two degrees of freedom, integrating a genetic algorithm to compensate for the system’s feedforward loop [26,27]. However, genetic algorithms have a relatively long response time, and the method for designing control parameters is complex [28,29]. Traditional sliding mode control is prone to chattering. In recent years, scholars such as A. Levant and Amjad J Humaidi have successively proposed high-order sliding mode control (HOSM) and adaptive backstepping sliding mode control based methods [30,31]. While effective in suppressing resonance, this advanced algorithm-based approach involves substantial computational demands and is complicated by its inherent complexity, making it challenging to implement given the hardware constraints of today’s servo systems.

**Figure 3:**
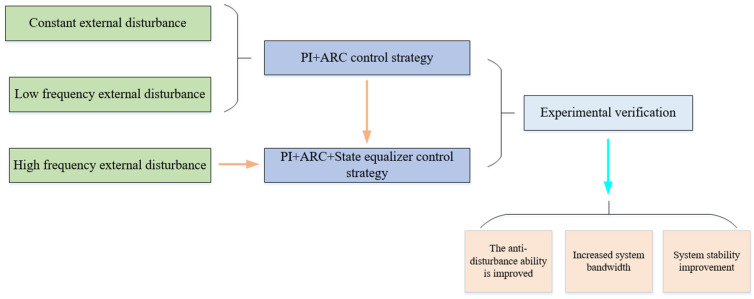
Overall scheme design diagram.

A key issue of the aviation optoelectronic stabilization platform under external disturbances is the uncertainty of its parameters. Therefore, a PI + ARC control strategy has been proposed to address this issue. However, when the disturbance is a high-frequency disturbance introduced by mechanical resonance, the transient performance and robustness of the PI + ARC control strategy will be limited. Therefore, a PI + ARC + State equalizer control strategy is proposed for suppressing high-frequency disturbances in aviation optoelectronic stability platforms. The overall scheme design of this article is shown in Figure 3. This is a new control design with high practical engineering application value. The contribution of this study can be emphasized as follows:

1. By proposing a PI + ARC + State equalizer control strategy, the impact of high-frequency disturbances on the aviation optoelectronic stability platform is reduced, the system bandwidth is increased, and the stability of the system is improved.
2. It has been proven that the PI + ARC control strategy can effectively compensate for fixed or low-frequency disturbances, but its compensation effect is limited for high-frequency disturbances. PI + ARC + State equalizer control strategy has been proposed to address the shortcomings in this control aspect.
3. Bandwidth experiments, disturbance experiments, and robustness experiments were conducted using an actual aviation optoelectronic stabilization platform. Further verification has been conducted that the PI + ARC + State equalizer control strategy can effectively suppress high-frequency disturbances caused by mechanical resonance, improve system bandwidth, and increase system stability compared to the PI + ARC control strategy.

## 2. Design of Adaptive Robust Algorithm Based on State Equalizer

This section initially establishes a simplified model of the aviation optoelectronic stabilization platform, elucidating the impact of high-frequency disturbances due to mechanical resonance on the actuator’s output torque, which in turn destabilizes the control system of the aviation optoelectronic platform. Subsequently, it elucidates the influence of high-frequency disturbances on the resilience of adaptive robust control algorithms. Finally, based on the adaptive robust control algorithm, a state equalizer speed closed-loop is integrated into the control system. Analysis of the transfer function of this new closed-loop control system demonstrates that it effectively mitigates the impact of high-frequency disturbances on the controller bandwidth. This enhancement not only ensures system robustness but also bolsters the anti-interference capacity of the aviation optoelectronic stabilization platform.

### 2.1. Analysis of the Impact of Mechanical Resonance on the Anti-Interference Ability of Aviation Optoelectronic Stability Platforms

The servo system of the aviation optoelectronic stability platform comprises three loops, from the outside to the inside: position loop, speed loop, and current loop. The position loop primarily facilitates tracking of targets, where the position refers to the relative position between the camera and the carrier, rather than the inertial space position; The visual axis stabilization primarily relies on the speed loop, which ensures tracking and control performance. The speed loop precisely replicates or follows a process, directly influencing the dynamic characteristics and disturbance resistance of the aviation optoelectronic stabilization platform; The primary purpose of the current loop is to ensure that the current precisely tracks voltage fluctuations, thereby accurately controlling the motor’s output torque. This loop effectively mitigates disturbances such as back electromotive force and electronic noise, simplifies the motor’s model, and facilitates the design of servo controllers by providing straightforward and intuitive conditions.

**Figure 4:**
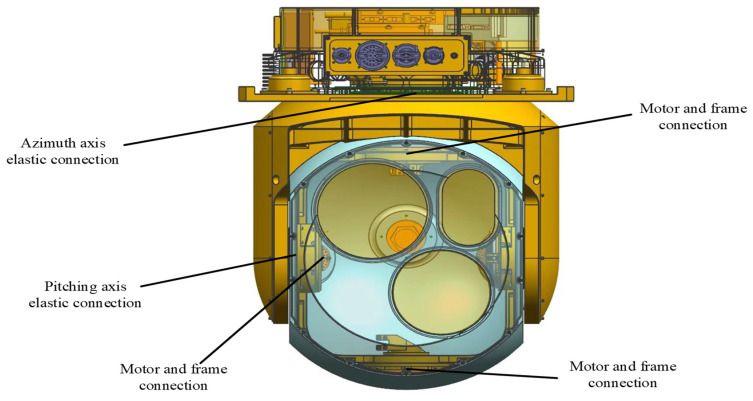
Elastic Position Distribution of Aviation Optoelectronic Stability Platform.

The research object of this article is a two axis and two frame aviation optoelectronic stability platform. The servo drive system’s transmission devices typically consist of a transmission shaft system, coupling, and reducer. Ideally, the transmission device is considered a rigid body, but absolute rigidity does not exist. As the demands for the visual axis accuracy of aviation optoelectronic stabilization platforms increase, the elastic properties of actual transmission devices can no longer be ignored. The elastic division diagram of the aviation optoelectronic stabilization platform is shown in Figure 4. When the rotational inertia of the load is relatively large and the transmission device has high elasticity, the system is prone to mechanical resonance, leading to high-frequency disturbances. If high-frequency disturbances are outside the bandwidth, they will not have a significant impact on the system, however, the controller gain will be relatively low, limiting the system’s dynamic performance; If the system bandwidth is relatively large, high-frequency disturbances may even fall within the bandwidth near the shear frequency, causing resonance and rendering the entire system inoperable.

For the convenience of further research on the controlled object. Simplify the structure of its control system to the one shown in Figure 4. Among them, $J_{M}$ is the motor side moment of inertia, $J_{L}$ is the load side moment of inertia, ${\dot{\theta }}_{M}$ is the motor speed, and *T* is the motor side output torque. The elastic impedance of the controlled object structure is composed of the viscous damper coefficient b and the stiffness coefficient *K*. $K_{R}$ represents a measurement device such as a gyroscope, $K_{A}$ represents an amplifier, and $K_{T}$ represents the torque constant of the motor.

**Figure 5:**
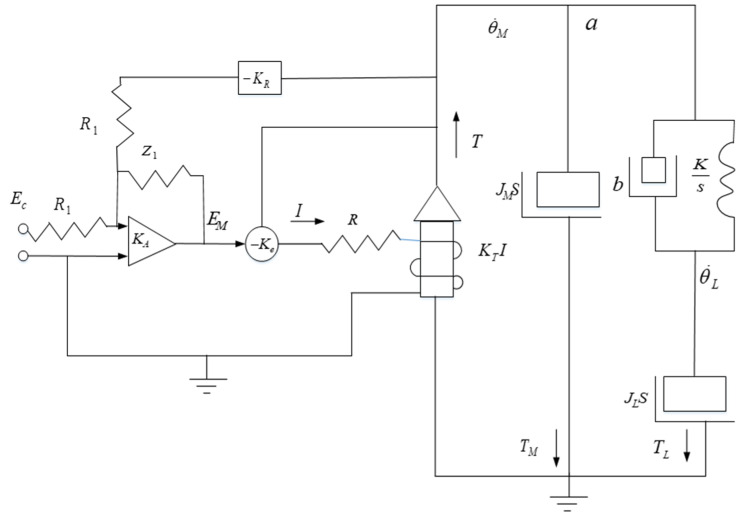
Simplified System of Aviation Optoelectronic Stability Platform.

The transfer function of the simplified system motor load impedance at point a in Figure 5 is:

(2) $$\frac{{\dot{\theta }}_{M}}{T}=M_{\left(s\right)}=\frac{1}{(J_{M}+J_{L})sF}$$

Among them, *F* is the resonance peak factor, and *F* can be expressed by the following equation:

(3) $$F=\frac{s^{2}/\omega_{R}^{2}+(b/k)s+1}{s^{2}/\omega_{AR}^{2}+(b/k)s+1}$$

Among them, $\omega_{AR}$ is the anti resonant frequency, which can be represented by Equation (4); $\omega_{R}$ is the resonant frequency, which can be represented by Equation (5).

(4) $$\omega_{AR}={\left(\frac{K}{J_{L}}\right)}^{1/2}$$

(5) $$\omega_{R}={\left[\frac{K}{\left(J_{M}J_{L})(J_{M}+J_{L}\right)}\right]}^{1/2}$$

According to Equation (4), $\omega_{AR}$ is mainly related to the structural stiffness of the aviation optoelectronic platform and the rotational inertia of the motor load. According to Equation (4), $\omega_{R}$ is related to the structural stiffness of the aviation optoelectronic platform, the rotational inertia of the motor load, and the rotational inertia of the load. The larger the stiffness coefficient *K* of the system, the better the structural stiffness of the aviation optoelectronic platform, the higher the mechanical resonance frequency, and the further increase in the system bandwidth. Due to the same viscous damping parameter terms for $\omega_{R}$ and $\omega_{AR}$, when Equation (2) is a standard quadratic form, the expression for damping ratio is as follows:

(6) $$\frac{b}{k}=\frac{2\zeta_{R}}{\omega_{R}}=\frac{2\zeta_{AR}}{\omega_{AR}}$$

The resonant damping ratio coefficient can be obtained from Equation (6) as shown in Equation (7):

(7) $$\zeta_{R}=\frac{\omega_{R}}{\omega_{AR}}\zeta_{AR}$$

**Figure 6:**
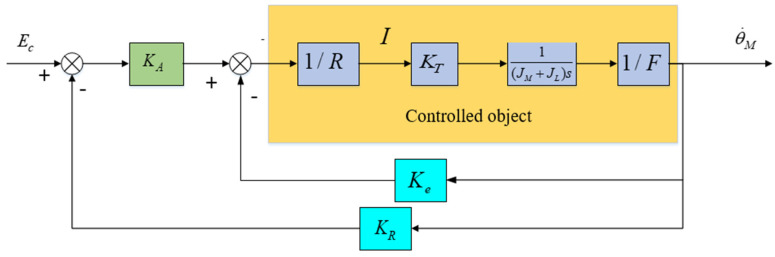
Control system closed-loop resonant loop.

According to Equation (7), it can be seen that the amplitude of the resonant peak point attenuates more than that of the anti-resonant peak point. Therefore, the block diagram of the closed-loop control system is shown in Figure 6, and its transfer function is:

(8) $$\frac{{\dot{\theta }}_{M}}{E_{C}}=\frac{K_{CL}}{F(s/\omega_{M})+1}$$

In the formula, the resonant closed-loop gain is $K_{CL}=K_{A}K_{M}/(1+K_{A}K_{M}K_{P})$, the closed-loop cutoff frequency of the optoelectronic platform is $\omega_{M}=\omega_{m}(1+K_{A}K_{M}K_{P})$, the open-loop cutoff frequency of the optoelectronic platform is $\omega_{M}=\frac{K_{e}K_{T}}{R(J_{L}+J_{M})}$.

**Figure 7:**
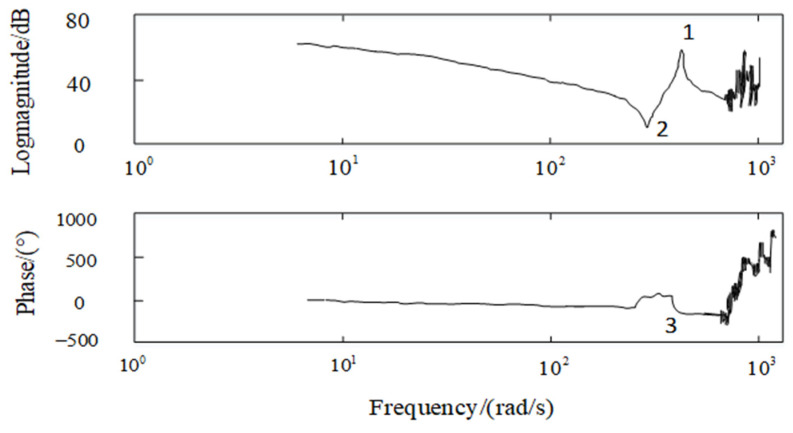
Scanning curve of platform model.

Given the input voltage signals of different frequencies on the azimuth axis of the aviation optoelectronic stability platform, the gyroscope data acquired from the experiments are processed using Matlab’s (2014.x) ident toolbox, resulting in the amplitude and phase frequency characteristic curves of the structural model of the aviation optoelectronic stabilization platform, as depicted in Figure 7.

According to the amplitude frequency and phase frequency characteristics of the controlled object, it can be seen that there is mechanical resonance in the controlled object. Among them, 312.37 rad/s is the anti resonance point 2 of the photoelectric platform, and 467.44 rad/s is the resonance point 1 of the photoelectric platform. At the same time, between points 1 and 2, the phase of the mathematical model of the photoelectric platform also undergoes a sudden change, as shown at point 3 in Figure 7.

The two resonant peaks mentioned above have a significant impact on the closed-loop performance of the optoelectronic platform. Firstly, the presence of resonant peaks can lead to inaccurate identification of control system model parameters, which in turn makes it difficult to accurately design controllers. Then, in the closed-loop loop, the sudden changes in system gain and phase near points 1 and 2 cause the system to oscillate, which in turn affects the robustness of the system. Therefore, adopting appropriate methods to suppress mechanical resonance is of great significance for control system design.

### 2.2. Adaptive Robust Control

Adaptive robust control is a high-performance robust control strategy introduced by Yao and Tomizuka to tackle parameter uncertainty disturbances in systems. This control method seamlessly integrates adaptive control and deterministic robust control design. Employing robust feedback techniques for the robust component, adaptive robust control effectively mitigates the impact of model uncertainties, including both parameter and non-parameter uncertainties. The ARC method addresses the suboptimal transient performance of the adaptive component and sidesteps the issue of asymptotic convergence that plagues robust control in the presence of parametric uncertainty alone, thereby meeting the application demands of aviation optoelectronic stabilization platforms.

In the research process of this method, the following assumptions were proposed

**Assumption 1.**  *The framework and shaft system of the aviation optoelectronic stability platform are rigidly connected.*

According to Assumption 1, the stiffness coefficient K→∞, resonance peak factor F→1, load inertia, and motor inertia are equal, meaning $J_{M}=J_{L}$. Consequently, the inertia is denoted by $J_{M}$. Hence, Equation (2) is simplified as follows:

(9) $$\frac{{\dot{\theta }}_{M}}{T}=M_{\left(s\right)}=\frac{1}{J_{M}s}$$

**Figure 8:**
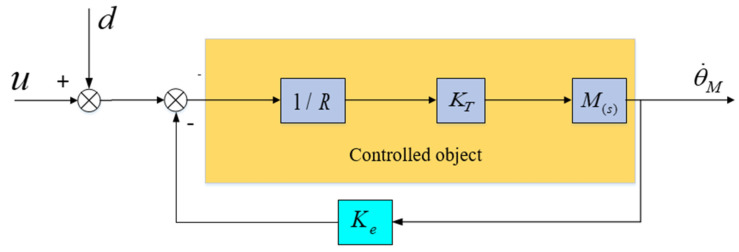
Simplified model diagram of aviation optoelectronic platform.

The aviation optoelectronic stabilization platform is simplified to the model depicted in Figure 8, where u represents the equivalent input voltage value in the system. d represents the voltage equivalent to the “total disturbance” in the system, referred to as the “equivalent disturbance voltage”. This voltage can replicate the effects of all disturbances in the system, including friction and mass imbalance disturbances.

Convert torque into motor input current to represent:

(10) $$T=K_{T}I$$

Given the inclusion of a current loop within the control system, the influence of the motor’s back electromotive force can be disregarded. Here, I represents the armature current.

(11) $$T=\frac{K_{T}}{R}(u+d)$$

Furthermore, Equation (2) can be transformed into:

(12) $$\frac{K_{T}}{R}(u+d)=J\frac{d{\dot{\theta }}_{M}}{dt}$$

Performing Laplace transform on Equation (12) yields:

(13) $$\frac{K_{T}}{R}(u+d)={\dot{\theta }}_{M}J_{M}s$$

(14) $${\dot{\theta }}_{M}=\frac{K_{T}}{J_{M}sR}(u+d)$$

If $J=J_{M}sR/K_{T}$, the simplified model of the aviation optoelectronic stability platform system is shown in Equation (15):

(15) $$G_{c(s)}=\frac{1}{Js}$$

Furthermore, the relationship between input and output is shown in Equation (16):

(16) $$J{\dot{\theta }}_{M}=u+d$$

The core idea of robust control is to construct an input μ, so that the relationship between input μ and output ${\dot{\theta }}_{M}$ in this case conforms to the nominal model:

(17) $$J_{n}{\dot{\theta }}_{M}=\mu$$

Among them, $J_{n}$ is the nominal model value. For simplicity, make the following assumptions.

**Assumption 2.**  *The difference between the nominal model and the true value is not significant, the impact on the system can also be ignored, which is* $J_{n}=J$.

The sliding mode variable P is defined as:

(18) $$P=\theta -\frac{1}{J_{n}}\int_{0}^{t}\mu_{\left(\tau \right)}d\tau$$

From Equations (16)–(18), it can be concluded that:

(19) $$J\dot{P}=u+d-\mu$$

The design control law is:

(20) $$u=u_{1}+u_{2}$$

$$u_{1}=-KP,\;u_{2}=u-\hat{d}$$

Among them, K is a positive definite constant. Assuming that $d_{M}$ and $d_{m}$ respectively perturb the upper and lower boundaries, there are:

(21) $$d\in (d_{m},d_{M})$$

After substituting Equation (20) into Equation (19), it can be concluded that:

(22) $$J\dot{P}+KP=-\tilde{d}$$

In the formula, $\tilde{d}=\hat{d}-d$ is the estimation error. If $\tilde{d}$ is considered as the input and P as the output, then Equation (22) can be considered as a first-order control system. The properties of first-order control systems suggest that: $\left|P_{\left(\infty \right)}\right|\leq \frac{{\tilde{d}}_{\left(\infty \right)}}{K}$, so as long as the value of K is increased as much as possible, the goal of reducing the value of P can be achieved, which is also a common strategy in robust control. However, in actual control systems, the presence of system chattering and mechanical resonance means that the value of K cannot be increased indefinitely. When the value of K increases to the upper limit, the size of the value of P will depend on the size of $\tilde{d}$. In order to further improve the performance of the system, an adaptive law is introduced for the estimation of $\hat{d}$ to reduce the value of estimation error $\tilde{d}$.

The expression for the adaptive law is:

(23) $$\dot{\hat{d}}=proj_{d(\Gamma P)}=\{\begin{array}{cc} 0, & \;\mathrm{if}\;\{\begin{array}{c} \hat{d}=d_{M}\;\mathrm{and}\;P>0\\ \hat{d}=d_{m}\;\mathrm{and}\;P<0 \end{array}\\ \Gamma P, & \;\mathrm{otherwise} \end{array}$$

**Figure 9:**
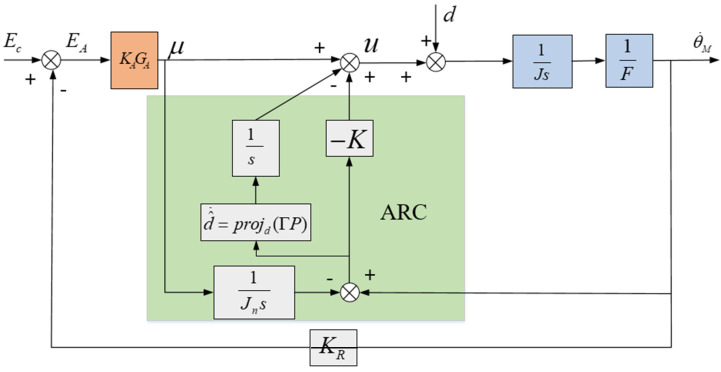
Control System with Adaptive Robust Control.

Based on the formula analysis, it is evident that if external disturbance d is constant, then regardless of the value of K, the adaptive robust controller can utilize an adaptive algorithm to cause $\hat{d}$ to approach d infinitely, ultimately eliminating the estimation error $\tilde{d}$. This, in turn, makes P=0, thereby mitigating the disturbance’s impact. If external disturbance d varies in real-time, it is essential to augment adaptive coefficient Γ to broaden the bandwidth of the resulting controller to align with the rate of change of d. However, as depicted in Figure 9, when the controlled object exhibits a resonance peak factor F, the aforementioned analysis suggests a scenario where the resonance frequency is relatively low and the resonance peak is relatively high. This can introduce high-frequency disturbances, thereby limiting the bandwidth of the control system. Therefore, the adaptive coefficient Γ cannot be infinitely increased, thus limiting the potential for further enhancing the transient performance and robustness of the control system.

In conclusion, the primary emphasis of this study is on enhancing the ability to suppress high-frequency disturbances in aviation optoelectronic stabilization platforms, thereby improving the control system’s bandwidth and robustness.

### 2.3. State Equalizer

Based on the analysis above, it is evident that high-frequency disturbances stemming from mechanical resonance in the aviation optoelectronic platform limit the further enhancement of the control system bandwidth, thereby impacting the dynamic performance of adaptive robust control methods. Therefore, in the closed-loop control loop of Figure 6, it is proposed to add state equalizer speed closed-loop control, which will generate a balanced speed response and reverse compensate to the control system. By using this method, the amplitude variation of the input signal can be ignored, and the speed and torque response characteristics of the motor can be kept stable within the resonant frequency range, thereby ensuring the smoothness of the frequency response of the control system.

**Figure 10:**
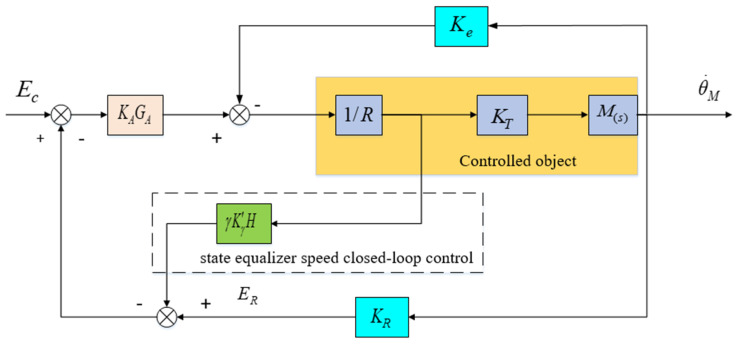
Resonant balanced speed closed-loop system.

The design approach for the state equalizer involves adding a resonant balancing speed loop, as depicted in Figure 10, to the closed-loop resonant loop depicted in Figure 5. This is done to mitigate the impact of mechanical resonance on the stability of the aviation optoelectronic stabilization platform control system. Once the state equalizer is designed, it can be integrated back into the control system to correct the model and suppress resonance.

After adding a state equalizer velocity loop, its closed-loop response characteristics can be represented by the difference between the voltage signal $E_{R}$ corresponding to the velocity information collected by the gyroscope and the armature current I. According to the schematic diagram in Figure 10, the transfer function of the state equalizer speed closed-loop system can be obtained as follows.

(24) $$\frac{{\dot{\theta }}_{M}}{E_{C}}=\frac{K_{A}K_{M}G_{A}}{\left(s/\omega_{m})F[1-(\gamma K_{\gamma }H)(K_{A}G_{A}/R)]+(1+K_{A}K_{M}K_{R}G_{A}\right)}$$

As shown in Figure 6, The expression for the current response I in the high-frequency disturbance closed-loop of the aviation optoelectronic stability platform is shown in Equation (25):

(25) $$\frac{I}{E_{C}}=\frac{(K_{A}/R)s}{s+\omega_{M}/F}$$

**Figure 11:**
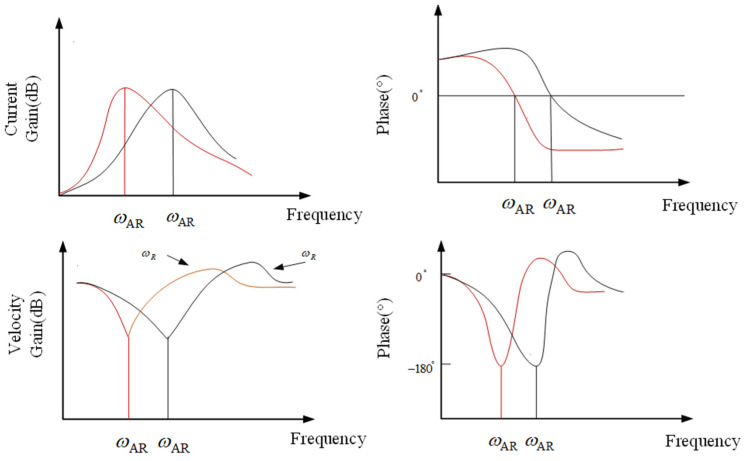
Curve plot of the influence of current response and motor speed on the closed-loop.

Based on Equations (8) and (25), the frequency response Bode diagram for the motor speed and current functions is depicted in Figure 11, facilitating the analysis of the variability between the two variables.

The current response reaches its maximum value at $\omega_{AR}$ and is consistent with the phase of $E_{C}$. In addition, the speed of the motor will rapidly decrease at point $\omega_{AR}$, and the phase difference with $E_{C}$ is 180°. Within the limited range of $\omega_{AR}$, when the speed amplitude of the motor is the smallest, the amplitude of the current response is the maximum value. In this scenario, despite the presence of an anti-resonance point, the phase difference between the resonance and anti-resonance points exerts only a modest constraint on the amplitude of the resonance point. In conclusion, the presence of the resonant peak factor F significantly affects the performance of the control system.

Comparing Equations (8) and (24), it can be seen that in the denominator term, the parameter term of the state equalizer can be used to offset the influence of the resonant peak factor F on the control system. Therefore, Equation (26) can be used as an expression for the closed-loop parameter term of state equalizer.

(26) $$K_{\gamma }H=\frac{R}{K_{A}G_{A}}$$

And: the coefficient of the state equalizer is γ=1.

The closed-loop transfer function of the control system can be rewritten as follows:

(27) $$\frac{{\dot{\theta }}_{M}}{E_{C}}=\frac{K_{A}K_{M}G_{A}}{1+K_{A}K_{M}K_{R}G_{A}}$$

**Figure 12:**
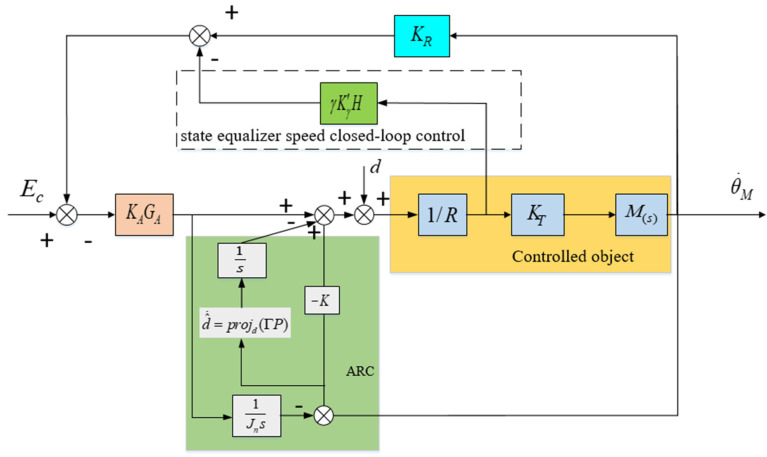
Principle diagram of control structure combining adaptive robust control method and state equalizer.

According to the resonant balancing speed closed-loop circuit in Figure 10, in order to ensure the stability of the current loop, the coefficient γ of the state equalizer in Equation (24) cannot be greater than 1. Therefore, the value of γ needs to be adjusted between 0 and 1 based on the magnitude of high-frequency disturbance. When γ→1, the resonance peak factor F in Equation (24) is cancelled out, and thus the mechanical resonance frequency $\omega_{m}$ is also eliminated. At this point, the closed-loop bandwidth of the system is mainly determined by amplifier system $G_{A}$, which can eliminate the impact of high-frequency disturbances caused by mechanical resonance on the control system. The control structure after combining the adaptive robust control method with the state equalizer is shown in Figure 12. After combining the two methods, the state equalizer algorithm can increase the bandwidth of the closed-loop system, which can further increase the gain K value, thereby improving the anti-interference effect and robustness of the control system, and ultimately achieving the goal of improving the visual axis stability accuracy of the aviation optoelectronic stability platform.

## 3. Experimental Verification

**Figure 13:**
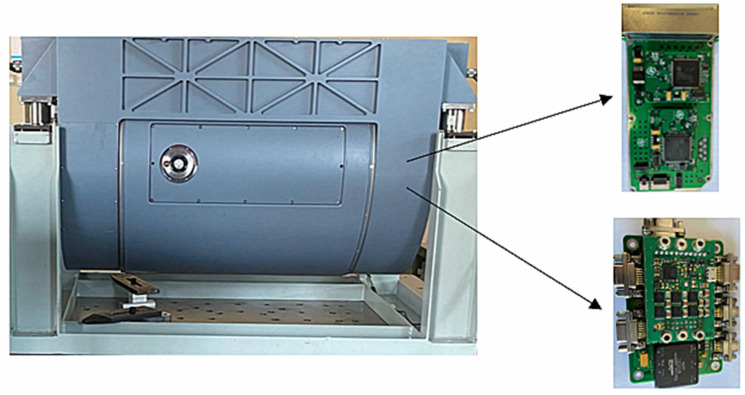
Experimental platform.

Taking the aviation optoelectronic stabilization platform shown in Figure 13 as the control object. The experiment is conducted on the azimuth axis of the aviation optoelectronic stability platform. In order to comprehensively test the compensation algorithm performance of PI + ARC using a state equalizer speed closed-loop, this paper conducts bandwidth testing experiments, disturbance suppression ability experiments, vibration experiments, and controller robustness adaptability experiments. As a comparison, the control method of PI + ARC also underwent the above experiments.

### 3.1. Bandwidth Test

**Figure 14:**
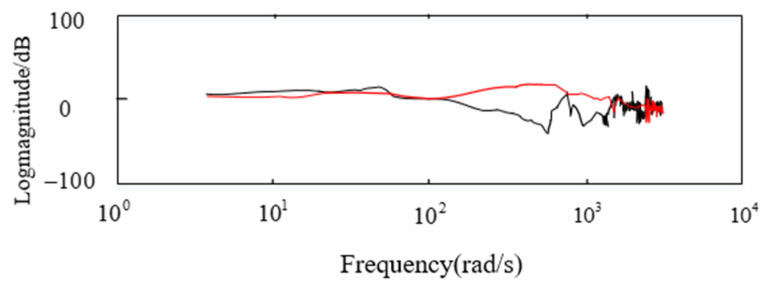
Bode diagram before and after compensation.

After compensating the control system of the aviation optoelectronic stability platform using the state equalizer speed closed-loop, the bandwidth of the state equalization speed closed-loop is tested on the basis of PI + ARC and PI + ARC. Figure 14 shows the amplitude frequency characteristics of the control system before and after compensation.

Figure 14 shows the closed-loop bandwidth test curves of the system under two different states. The red color in the figure represents the amplitude frequency characteristics of the PI + ARC + State equalizer algorithm, while the black color represents the amplitude frequency characteristics of the PI + ARC algorithm. Moreover, as shown in Figure 12, the bandwidth of the PI + ARC algorithm is 27.22 Hz, while the bandwidth of the PI + ARC + State equalizer algorithm is 40.19 Hz Compared with the PI + ARC algorithm, the PI + ARC + State equalizer algorithm has an improvement of 47.6% in bandwidth.

### 3.2. System Anti-Interference Ability Experiment

#### 3.2.1. Speed Disturbance Experiment

**Figure 15:**
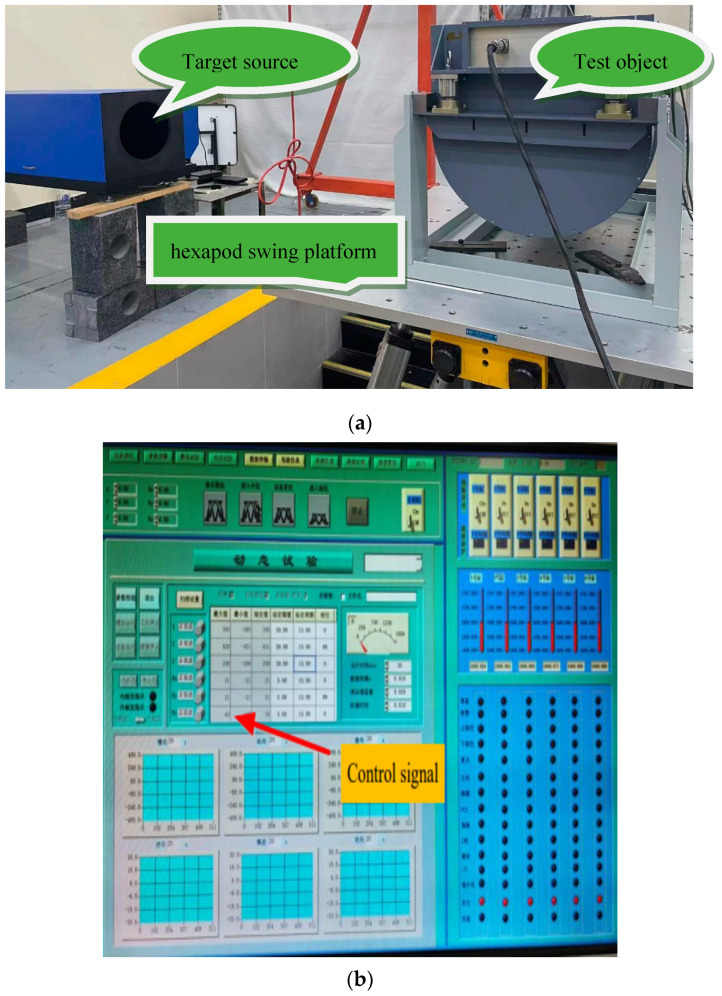
Method of experimental equipment for system anti-interference ability. (**a**) Installation diagram for the aviation optoelectronic stabilization platform for anti-interference testing. (**b**) Upper computer control interface for the experimental platform.

To verify the compensation effect for high-frequency disturbances caused by the structural stiffness of the aviation optoelectronic stability platform before and after adding the state equalizer algorithm to the controller. The aviation optoelectronic stability platform is installed on a high-frequency hexapod swing platform to simulate flight status. The detailed experimental procedure is depicted in Figure 15. The non English characters in Figure 15 are the upper computer interface for setting disturbance parameters written by the high-frequency hexapod swing platform software manufacturer.

**Figure 16:**
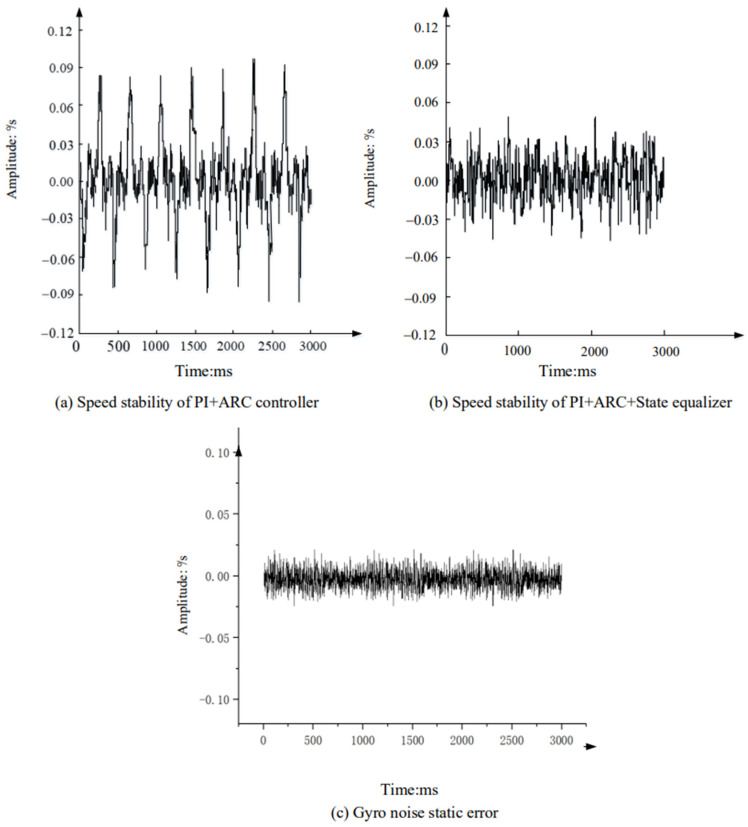
Comparison of speed stability experiments.

In the experiment, the aviation optoelectronic stabilization platform is set to an expected rotational speed of zero. With the hexapod swing platform undergoing sinusoidal motion at any frequency within 2 Hz, the gyro signal is measured to assess the platform’s speed loop’s ability to isolate disturbances. Figure 16 illustrates the speed stability and static error of gyro noise when the swing platform undergoes sinusoidal motion at 1°2 Hz, using the PI + ARC controller and PI + ARC + State equalizer controller, respectively.

From Figure 16 analysis, it can be seen that compared with the PI + ARC controller, the platform speed is stable and significantly improved after adding a state equalizer speed closed-loop, with a peak value of approximately 0.04°/s. Considering the high noise level of the gyroscope, the peak noise level of the gyroscope is 0.02 when the platform is absolutely stationary. This indicates that in such a noisy system, the improvement limit of this system is 0.02, indicating that the disturbance suppression results of the optoelectronic stable platform with the PI + ARC control algorithm incorporating state equalizer speed closed-loop are very satisfactory.

**Figure 17:**
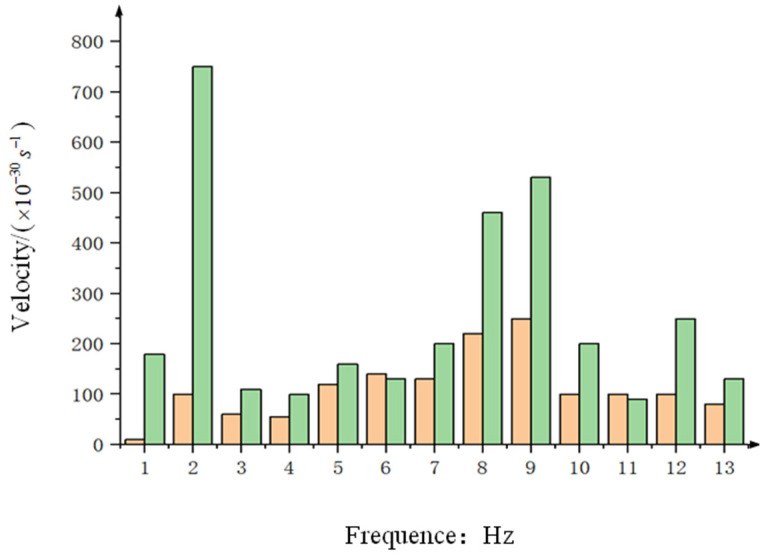
Under the disturbance of 2 Hz, the Fourier transform of the internal frame rate before and after the state equalizer speed closed-loop is adopted.

After analyzing the spectra of the data in Figure 16a,b, the results are depicted in Figure 17 (orange represents the data with a state equalizer speed loop added to PI + ARC, while green represents the data only using PI + ARC). Upon comparison, it is evident that by incorporating a state equalizer speed loop into the PI + ARC system, the residual disturbance at 2 Hz is approximately 2/15 that of the optoelectronic platform using PI + ARC alone, resulting in a 17.50 dB improvement in speed disturbance isolation.

Table 1 shows the extent to which the state equalizer compensates for the improvement of velocity disturbance isolation by the platform before and after the hexapod swing platform swings at a step frequency of 0.5 Hz to 2 Hz with an amplitude of 1°. As shown in the table, at different oscillation frequencies, the addition of state equalizer speed closed-loop in the control system significantly improves the isolation degree of the platform from speed disturbances.

#### 3.2.2. Target Tracking Experiment

**Figure 18:**
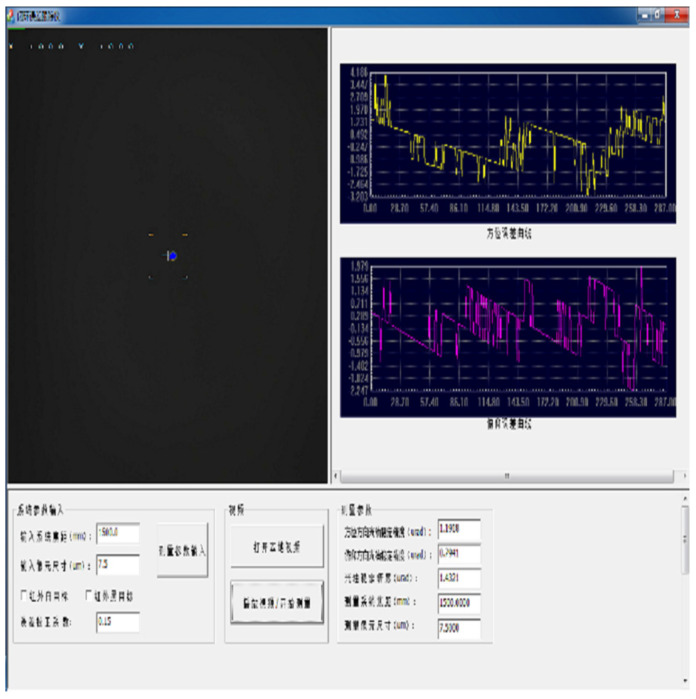
Image Analysis System.

To compare the application effect of disturbance compensation using the state equalizer algorithm with and without PI + ARC on the actual equipment of the aviation optoelectronic stabilization platform, the aviation optoelectronic stabilization platform was installed on a hexapod rocking platform. The specific details are shown in Figure 15. By analyzing the image analyzer shown in Figure 18, the shaking of the target relative to the line of sight can be obtained, allowing for a comparison of the line of sight stability accuracy of the two axis and two frame aviation optoelectronic stabilization platform. The non English characters in Figure 18 are the upper computer interface written by the software manufacturer to display the visual axis accuracy parameters.

**Figure 19:**
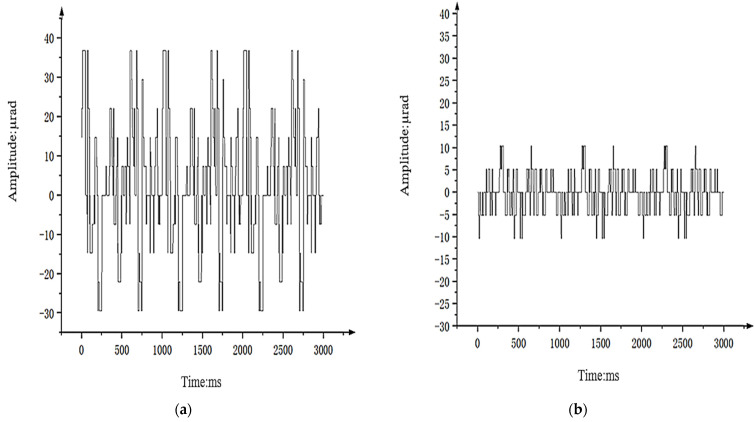
The shaking range of the Los of the controller. (**a**) shaking range of the Los of PI + ARC controller; (**b**) shaking range of the Los of PI + ARC + State equalize controller.

Figure 19 shows the stability accuracy data of the airborne optoelectronic stabilization platform obtained by measuring the off target distance data of the platform when it operates in visible light TV tracking mode and the hexapod swing platform moves in a sinusoidal motion with an amplitude of 1° and a frequency of 2 Hz. Figure 19a shows the off target information using PI + ARC + State equalizer controller, which is approximately ± 10 μrad, only 2/7 of Figure 19b. This indicates that the visual axis stability accuracy of the aviation optoelectronic stabilization platform has been further improved after the addition of state equalizer speed closed-loop.

### 3.3. System Vibration Experiment

In order to simulate the complex and ever-changing environment on the ground more realistically, in addition to the flight simulation turntable experiment mentioned above, vibration and impact experiments should also be conducted on the aviation optoelectronic stability platform to test its adaptability to complex environments.

**Figure 20:**
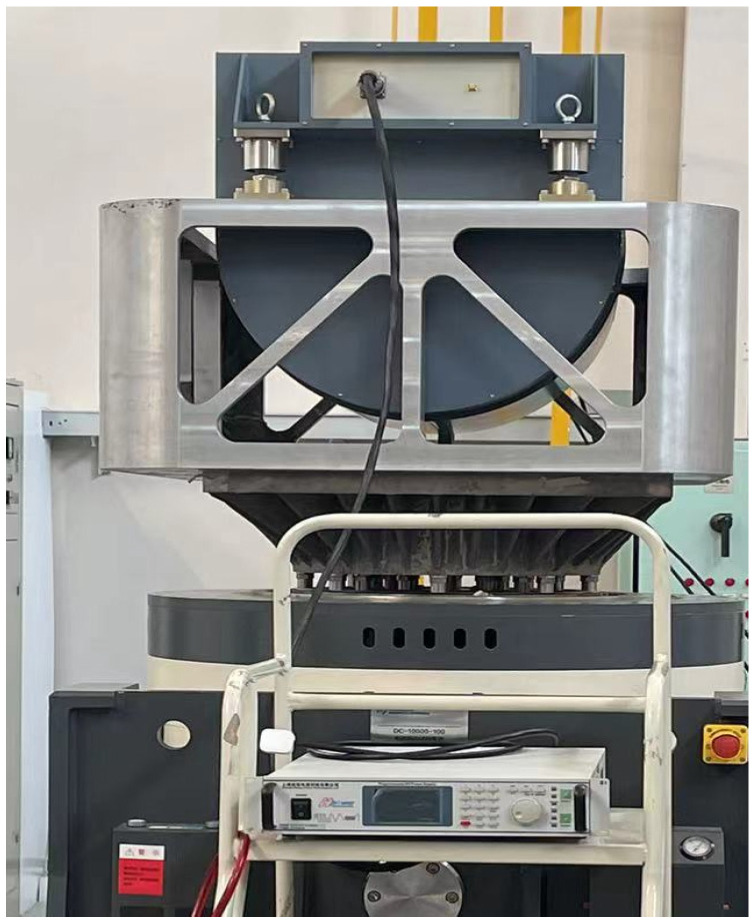
Vibration test device.

The experimental setup involves installing a two-axis, two-frame aviation optoelectronic stabilization platform system within a vibration testing device. This device facilitates random vibration tests to examine how the addition of a state equalizer to the PI + ARC controller impacts the suppression of mechanical resonance in the optoelectronic platform when structural changes occur due to variations in the platform’s structure. The specific operation is illustrated in Figure 20.

**Figure 21:**
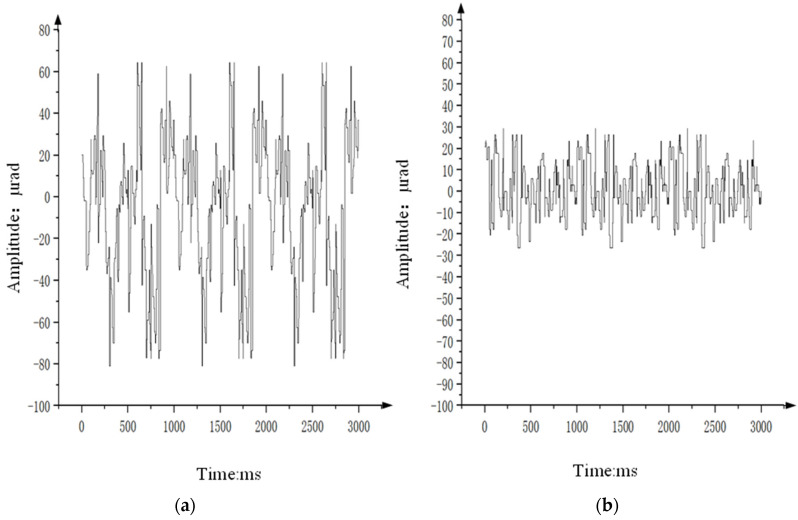
Shaking range of the controller’s Los. (**a**) shaking range of the Los of PI + ARC controller; (**b**) shaking range of the Los of PI + ARC + State equalize controller.

Using a vibration level of 3 *g*/Hz^2^ on the vibration table as an example, the aviation optoelectronic stabilization platform is fixed on the vibration table. The off-target information obtained under visible light load tracking is used to analyze the stability accuracy of the equipment’s visual axis. Figure 21 shows the off-target data information in the azimuth direction of the aviation optoelectronic stabilization platform when using PI + ARC controller and PI + ARC + State equalizer controller, respectively.

Table 2 shows the visual axis stability accuracy of the aviation optoelectronic stabilization platform under the condition of 2 *g*/Hz^2^–4 *g*/Hz^2^ vibration levels (the test results are also obtained from the image analyzer).

As shown in Figure 21a, the shaking range of the optoelectronic platform’s visual axis when using the PI + ARC control algorithm under vibration condition 3 *g*/Hz^2^ is represented by 38.11627 µrad. Figure 21b shows the visual axis shaking range of the optoelectronic platform using the PI + ARC + State equalizer control method under the same vibration conditions, which is only 1/3 of the PI + ARC algorithm.

The above results indicate that the PI + ARC + State equalizer control method has better adaptability to environmental changes. Table 2 shows the data on the visual axis shaking range using two different algorithms at 2 *g*/Hz^2^–4 *g*/Hz^2^ vibration levels, further proving that the PI + ARC + State equalizer control method exhibits stronger stability than the PI + ARC control algorithm in different environments.

### 3.4. System Robustness Experiment

With the controller unchanged, the model parameters of the control object are artificially altered to vary within 10%. The experiment from Section 3.2.1 is repeated to assess the system’s disturbance isolation when using a control strategy that integrates PI + ARC with a state balancer. Table 3 details the enhancement in disturbance isolation observed during the repeated velocity disturbance test in Section 3.2.1 when the model parameters vary within 10%.

**Figure 22:**
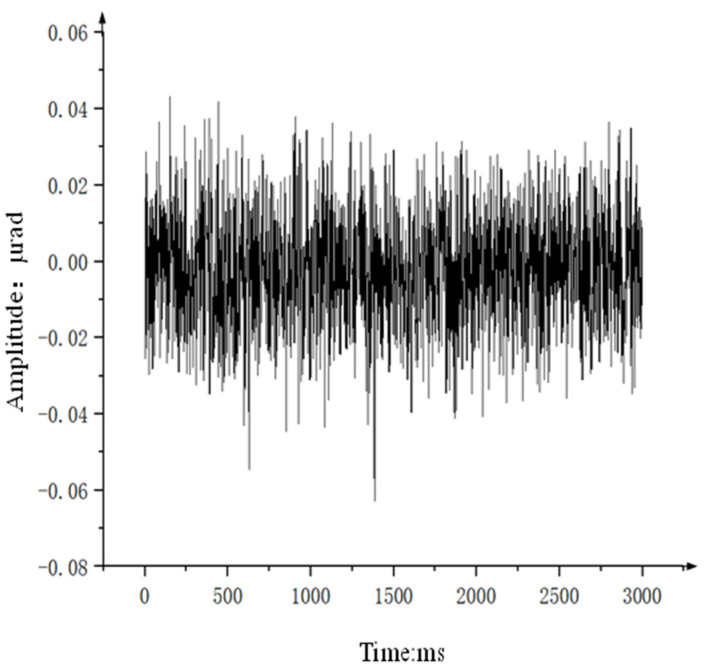
Gyroscopic Static Noise Data.

Next, a gyroscope with higher static noise is selected to repeat the comparative experiment in Section 3.2.1. The static noise of the gyroscope is shown in Figure 22, and Table 4 shows the specific data on the improvement of speed disturbance isolation before and after compensation using the state equalizer.

After performing Fourier transform on the feedback data of the gyroscope, it can be seen from Table 3 and Table 4 that the state equalizer speed closed-loop has reduced the isolation degree of velocity disturbance before and after compensation for the aviation optoelectronic stability platform control system. However, the control method of adding state equalizer speed closed-loop on the basis of PI + ARC still achieves better results than the PI + ARC control method. Therefore, the following conclusion can be drawn: adding a state equalizer speed closed-loop on the basis of the PI + ARC control method can improve the system’s anti-interference ability, while its robustness performance is basically consistent with the PI + ARC control strategy.

Drawing from the aforementioned experiments, the control algorithm that adds a state equalizer speed closed-loop to the PI + ARC framework, as proposed in this study, effectively suppresses mechanical resonance and enhances the control bandwidth. This approach not only ensures the robustness of the control system but also bolsters the aviation optoelectronic stabilization platform’s capacity to resist high-frequency disturbances. Consequently, the control algorithm of PI + ARC + state equalizer speed closed-loop fully meets the performance standards required for optoelectronic stabilization platforms in real-world applications.

## 4. Conclusions

PI + ARC control strategy is proposed to address the issue of parameter uncertainty in aviation optoelectronic stabilization platforms under external disturbances. However, when the disturbance is a high-frequency disturbance introduced by mechanical resonance, the fast convergence and stability of the PI + ARC control strategy will decrease. This article proposes a strategy of incorporating a state equalizer into a PI + ARC based control algorithm to suppress mechanical resonance. Compared to the current general PI + ARC control algorithm, adding a state equalizer speed closed-loop control system can better eliminate the impact of mechanical resonance on system bandwidth. The experimental results on the aviation optoelectronic stability platform show that after adding the state equalizer speed closed-loop, the system’s bandwidth has increased by 47.6%; The residual disturbance is only 2/15 of the PI + ARC control strategy; After changing the model parameters, it exhibits better stability. This indicates that the aviation optoelectronic stabilization platform can utilize the designed controller to possess both robustness and resistance to high-frequency disturbances, thereby meeting the practical application of the aviation optoelectronic stabilization platform in engineering.

At present, the control strategy based on PI + ARC + State equalizer has met the requirements of most control algorithms for high-frequency disturbance amplitude correction. However, the phase lag of using state equalizer speed closed-loop control strategy is relatively large, which will be a key research focus in the future.

## Figures and Tables

**Table 1:** The degree of improvement in the isolation degree of velocity disturbance by the platform before and after compensation.

Frequency/Hz	Increased Isolation Degree/dB	
	PI + ARC	PI + ARC + State Equalizer
0.5	7.18	13.47
1	9.71	15.42
1.5	10.23	16.38
2	9.94	17.50

**Table 2:** Comparison of visual axis stability accuracy of optoelectronic stabilization platform before and after compensation.

Vibration Level	PI + ARC	PI + ARC+ State Equalizer
2 *g*/Hz^2^	35.5018 µrad	13.0550 µrad
3 *g*/Hz^2^	38.11627 µrad	13.98008 µrad
4 *g*/Hz^2^	58.68746 µrad	19.87882 µrad

**Table 3:** The degree of improvement in disturbance isolation when the model parameters change by ±10%.

Frequency/Hz	Disturbance Isolation/dB Δ=−10%		Disturbance Isolation/dB Δ=10%	
	PI + ARC	PI + ARC+ State Equalize	PI + ARC	PI + ARC+ State Equalize
0.5	6.51	12.19	6.94	12.71
1	8.21	13.47	8.84	13.73
1.5	9.89	14.62	10.02	15.08
2.0	9.41	16.43	9.11	15.59

**Table 4:** The degree of improvement in the isolation degree of speed disturbance by the platform before and after the state equalizer speed closed-loop compensation.

Frequency/Hz	Disturbance Isolation/dB	
	PI + ARC	PI + ARC + State Equalizer
0.5	6.69	12.78
1	8.53	14.73
1.5	9.13	14.46
2	8.64	16.81

## Data Availability

Data are contained within the article.
